# Stream Hydrology Controls the Longitudinal Bioreactive Footprint of Urban-Sourced Fine Particles

**DOI:** 10.1021/acs.est.2c00876

**Published:** 2022-06-07

**Authors:** Jennifer D. Drummond, Susana Bernal, Warren Meredith, Rina Schumer, Eugènia Martí

**Affiliations:** † Integrative Freshwater Ecology Group, Centre for Advanced Studies of Blanes (CEAB-CSIC), Blanes, Girona 17300, Spain; ‡ School of Geography, Earth and Environmental Sciences, University of Birmingham, Edgbaston B15 2TT, U.K..; § Center for Geology and Environmental Cartography (Geocamb), University of Girona, Girona 17003, Spain; ∥ Division of Hydrologic Sciences, Desert Research Institute, Reno, Nevada 89512, United States

**Keywords:** fine particle standing stocks, aerobic respiration, streambed, stream metabolism, organic matter, urban point source, carbon cycle

## Abstract

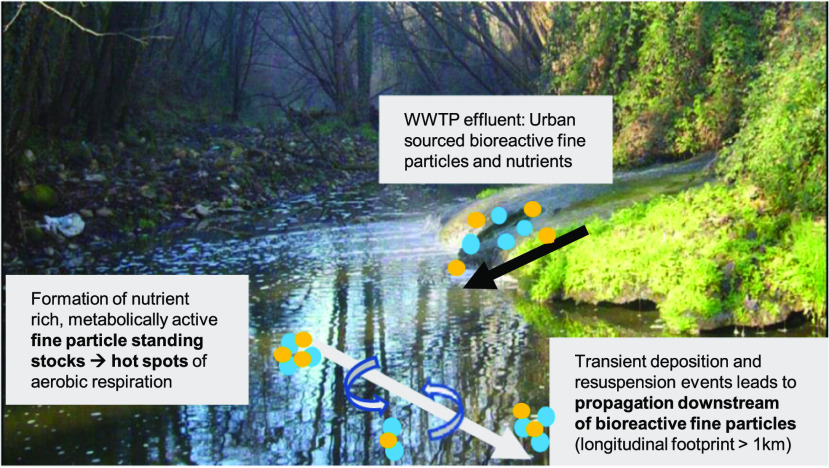

The relevance of wastewater treatment plant (WWTP) effluents in fluvial networks is increasing as urbanization grows in catchments. Urban-sourced fine particles from WWTP effluents deposit and accumulate in the streambed sediment of receiving streams over time and can fuel respiration rates, which can thus potentially increase rates of biogeochemical reactions and CO_2_ emissions to the atmosphere. We aimed to provide a quantitative assessment of the influence of WWTP-sourced fine particles deposited in the streambed sediment on stream metabolic activity for 1 year in an intermittent Mediterranean stream. More nutrient-rich and metabolically active fine particle standing stocks were observed downstream of the WWTP, propagating to the end of the 820 m study reach, especially during the dry period (*i.e.*, when the dilution capacity of the stream to WWTP inputs is <40%). Based on the longitudinal patterns of fine particle standing stocks and their metabolic activity, we estimated that the in-stream bioreactive capacity associated with these fine particles could potentially lead to substantial carbon dioxide emissions to the atmosphere (3.1 g C/m^2^/d). We show the importance of incorporating fine particle standing stocks downstream of point source inputs, particularly WWTPs in intermittent streams, into carbon budgets.

## Abstract 2

Urban-sourced bioreactive fine particles form hot spots of aerobic respiration, with direct implications to stream metabolism and global carbon cycles.

## Introduction

Approximately 0.8 peta-grams of particulate organic matter (POM) enters streams and rivers annually from terrestrial sources,^1−3^ which is processed by stream biota from headwaters toward the ocean, as described by the river continuum concept.^4^ As stream size increases from upstream to downstream ecosystems, internal processing of carbon (C) sources becomes relatively more important,^5,6^ leading to potentially high rates of carbon dioxide (CO_2_) produced within streams and rivers. However, disruptions of the longitudinal continuum of C cycling along fluvial networks due to urbanization within the catchment are not included in the conceptual model of the global fluvial carbon budget. Although wastewater treatment plants (WWTPs) reduce the pervasive effects of urban and industrial activities on stream ecosystems, they are still important point sources of nutrients, organic matter (OM), fine particles, and microbes to streams.^7−9^ Previous studies have demonstrated an increase in stream metabolic activity, in particular ecosystem respiration, downstream of WWTP effluent inputs, which has been broadly linked to increased nutrients and microbial abundance.^10−12^ While large particles are preferentially removed by filtration during the wastewater treatment process, fine particles (*i.e.*, <100 μm) can pass through the filters and discharge into receiving streams. Bacteria preferentially attach to the finer fraction of particles,^13^ and total bacterial abundance and biomass are highest in the finest sediment fraction (<63 μm), despite being a small percentage of the total sediment.^14^ Furthermore, the fraction of fine particulate organic matter (FPOM, size <1 mm) serves as an important source of energy and nutrition to in-stream biota because it has a high surface area-to-volume ratio that promotes microbial colonization and subsequent mineralization, decomposition of OM, nutrient cycling, and formation of new biomass.^15−17^ As human populations tend to concentrate in urban areas worldwide,^18^ effects of urban development on fluvial networks can become more commonly prevalent within catchments. In this context, urban point sources can exert a relevant influence on the physical and chemical characteristics of receiving streams; however, their effects on functional aspects, such as biogeochemical processes, are still widely unknown (but see Merseburger *et al.*(19) and Burdon *et al.*(20)).

The flow regime is a master variable that modulates the influence of WWTP effluent inputs on stream ecosystems, given that natural discharge is extremely variable and WWTP outflows are relatively constant.^21^ First, the relative contribution of WWTP effluent inputs to stream discharge increases with decreasing dilution capacity of the receiving stream (*i.e.*, lower stream discharge^22^). Second, the flow regime influences the transport and retention of particles, and therefore, whether FPOM will be transported downstream (*i.e.*, propagation) or deposited and stored close to the source input. FPOM is transported downstream through a series of transient immobilization and remobilization events that encompass a wide range of characteristic retention times dependent on the hydrological conditions.^23,24^ FPOM is physically retained in stream transient storage areas, such as streambeds, that are susceptible to biotic interactions and then processed biologically.^16^ For instance, FPOM deposited within the streambed sediment can remain for hours to months close to the input source and become a long-term source of carbon.^25^ Therefore, controlling factors and mechanisms of FPOM storage, transport, and transformation are all important to understand FPOM dynamics along stream networks.^24,26^ Streambeds are exposed to a high influx of oxygen, nutrients, and fine particles due to stream water to groundwater exchange, especially at the top few centimeters. These hydrological interactions influence fine particle standing stocks, which are a highly reactive and dynamic compartment most easily influenced by varying hydrology.^27−30^ While the river continuum concept predicts how OM processing changes longitudinally with changing physicochemical and biological drivers, the balance between OM deposition and accumulation *vs* transport is still not well understood. As such, information on the overall impact of fine particles accumulated within streambed sediments, particularly downstream of point sources, on the global carbon cycle is still lacking.

**Figure 1:**
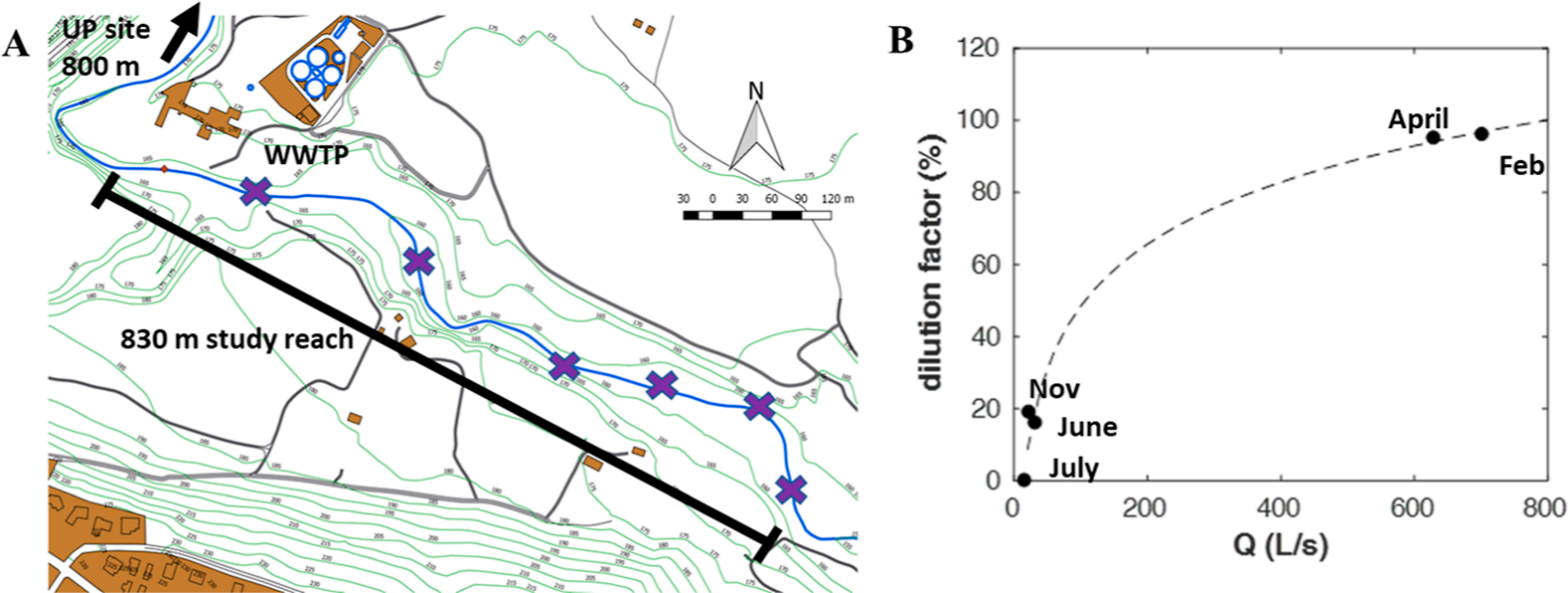
Sampling locations and hydrological conditions (*i.e.*, DF) during the 1 year field study. (A) Site map with sampling locations in La Tordera River located near the village of Santa Maria de Palautordera (NE Spain, lat 41°41′3.47″ N, long 2°27′33.19″ W). (B) Relationship between the DF and stream discharge (*Q*) based on the five sampling dates. Slotted line shows the best-fit of this relationship (DF = 24.8*ln(*Q*) – 65.7, *r*^2^ = 0.99, and *p* < 0.05).

The objective of this study was to provide a quantitative assessment of the influence of WWTP effluent inputs on the bioreactive capacity of fine particles deposited in the streambed sediment. We aimed to understand the influence of fine particle standing stocks on stream heterotrophic metabolism and on stream C cycling. While previous studies mostly analyze fine particle standing stocks upstream and downstream of a point source,^10,11^ we sampled systematically from where mixing with the WWTP effluent was achieved at 100 to 830 m downstream of the WWTP point source. In this way, we could assess how fine particle standing stocks and their biogeochemical characteristics distributed longitudinally along the stream. We analyzed fine particle standing stocks upstream and downstream of a WWTP effluent input in La Tordera (Figure 1A), an intermittent Mediterranean stream over a wide range of hydrological conditions. The dilution factor (DF) was used as an indicator of the relative proportion of WWTP effluents to stream discharge (Figure 1B). Lower DF values indicate higher influence of WWTP effluent inputs, with dry and wet periods defined by the DF threshold of 40% based on previous work.^31^ We measured the standing stock quantity of total fine particulate matter (FPM) and the fraction of FPOM. We also characterized the quality of the FPM as the percentage of organic matter (% OM) and the relative proportion of nitrogen (% N) and % C. Moreover, we measured the microbial metabolic activity (MMA) associated with FPM using the resazurin-resorufin metabolic tracer system that was used as a proxy for aerobic microbial respiration.^32^ We expected FPM standing stocks to differ from upstream to downstream of the WWTP effluent inputs, with both FPM standing stock quantity and quality to depend on the DF, with more apparent differences during the dryer periods. Lastly, we expected the flow regime, as indicated by the DF, to alter the longitudinal distribution of FPM standing stock quantity and quality along the receiving stream reach.

## Methods

### Sampling Site

The field study site was located in the main course of La Tordera River, immediately downstream of the WWTP outlet of the village of Santa Maria de Palautordera (Catalonia, Spain). The WWTP (5808 population equivalents) performs biological secondary treatment with activated sludge. The discharge of the WWTP is relatively constant throughout the year (mean of 27.4 L/s). In contrast, the stream discharge (*Q*) can vary by several orders of magnitude within and between hydrological years. Thus, the contribution of the WWTP effluent to the total water of the receiving stream ranges from 3 to 100%.^33^ Samples were taken at six sites every ∼100 m in wadeable stream areas (*i.e.*, *x* = 100, 300, 530, 623, 720, and 830 m) along an 830 m long reach downstream of the WWTP outlet (Figure 1A) in order to examine the longitudinal pattern of fine particle accumulation within the streambed sediment (top 3 cm). When the stream was flowing upstream of the WWTP, we collected samples at an upstream site 800 m from the WWTP outlet. This site was used as a reference to evaluate the effect of the WWTP input. The distance of the first sampling location downstream of the WWTP effluent (*i.e.*, *x* = 100 m) was selected to ensure that the effluent was well mixed with the stream water on all sampling dates, regardless of hydrological conditions. Samples were taken on five dates in 2017 (8 February, 3 April, 13 June, 31 July, and 9 November), comprising a complete water year and accounting for seasonal effects with a wide range of hydrological conditions, which directly impact on the DF of the receiving stream (Figure 1B). June, July, and November were considered dry periods (DF <40%), and April and February were considered wet periods (DF >40%) (Figure 1B). On the July sampling date, upstream of the WWTP outlet was dry, whereas the stream was flowing upstream of the WWTP on the other four sampling dates.

### Field Methods

On each sampling date, we collected three replicates of FPM at each of the six downstream sampling locations. Similar locations were chosen on each sampling date. The upstream site consisted of two locations, each with three replicates. Sampling of FPM in the streambed followed a modified method of Petticrew *et al.*,^34^ which involved pushing a 35 cm diameter bucket into the stream bed to form a seal and isolate the flow of the surrounding water. The sampling depth was recorded (five replicates), and then, the top approximately 3 cm of the sediment was agitated by hand to resuspend the fine particles into the water column within the bucket. We set a 10 s settling period for the majority of the sand-sized sediment to settle out of the water, such that only material less than approximately 100 μm was sampled.^34^ A volume of stirred and well-mixed water containing suspended FPM was collected using a 1 L wide-mouth Nalgene bottle. This water sample was poured into vials, each of which was used for the analysis of a distinct variable described below in the laboratory methods. In June, July, and November, a stream water sample was also taken prior to any sediment disturbance at each sampling location. These samples were used as a reference of the quantity and quality of FPM in the stream water column. All samples were immediately placed on ice, protected, from sunlight, and kept in the fridge at 4 °C until analyzed.

For each sampling date, *Q* in L/s was estimated 200 m downstream of the WWTP point source using the velocity-area method. At this same site, the stream water level was continuously measured and calibrated with 10 discharge measurements following the velocity-area method to obtain a continuous reading of *Q* (L/s). The DF for each sampling date was calculated from the electrical conductivity measurement upstream (EC_UP_), at the WWTP point source (EC_WWTP_), and 100 m downstream of the point source (EC_DOWN_).
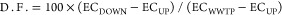
1

### Laboratory Methods

The FPM standing stock was estimated by filtering a known volume of each sample (∼100–200 mL) onto a pre-weighed 0.7 μm glass fiber filter. Filters were dried at 50 °C for at least 24 h to reach a dry stable weight and measured as FPM. Dry filters were then placed in the muffle furnace at 500 °C for 5 h and then at 50 °C for 24 h to measure FPOM. The % OM was calculated from FPOM/FPM × 100. To standardize the FPM and FPOM measurements at the different sampling locations, measured concentrations (g FPM or FPOM/mL) were converted to mass per stream surface area (g FPM or FPOM/cm^2^) by multiplying by the total water volume within the sampling bucket (mL) and then dividing by the surface area of the stream enclosed within the bucket.

Mass of C and N of FPM was determined using a CN analyzer (Thermo Finnigan Flash EA 1112, Waltham, MA, USA) and expressed as C/N molar ratios, % C and % N. Replicate samples were analyzed and averaged. The particle size of select fine particle samples was analyzed using a Mastersizer 2000 (Malvern Instruments Ltd., UK).

Rates of MMA (1/h) associated with FPM were estimated using incubations of collected samples with the Raz-Rru tracer.^32^ We poured a well-mixed 40 mL of the sub-sample of each collected FPM sample into a 50 mL vial. Vials were kept in a cool, dark place until the start of the incubation. In each vial, we added 400 μL of a Raz solution (0.022 g Raz/L), mixed well, and collected 4 mL of aliquots of the incubation at four time intervals during a 3 h incubation period (*t*_0_ = 15 min, *t*_1_ = 60 min, *t*_2_ = 105 min, and *t*_3_ = 165 min). During the incubation, the vials were shaken every 5 min to ensure homogeneous conditions in the mixed solution. The 4 mL of aliquots were filtered through a 0.7 μm pore-size glass fiber filter (GF/F, supplied by Whatman, UK). The first 1 mL was discarded, whereas the remaining 3 mL was placed in a vial and left under dark conditions to avoid light effects on Raz decay.

Immediately prior to Raz and Rru analyses, 0.3 μL of pH 8 buffer solution, generated by mixing 1 molar NaH_2_PO_4_·H_2_O with equal parts of 1 molar NaOH, was added to each sample vial. Raz and Rru concentrations were measured on a spectrofluorometer (Shimadzu/RF-5000, Kyoto, Japan) with wavelengths of 602 and 616 nm of excitation and emission for Raz and 571 and 585 nm of excitation and emission for Rru. Fluorescence readings (taken in triplicate and averaged per time interval) were converted to molar concentrations from a calibration curve (*r*^2^ = 0.99) using the same lot of Raz for all experiments.

The normalized turnover of Raz into Rru [*i.e.*, ln(Rru/Raz + *P*)] over incubation time was used to estimate the rates of MMA. Values of *P* indicate the production-decay ratio of Rru, which includes effects of irreversible sorption, photo decay, and any other mass losses of Raz and Rru. For the timescale of these incubations, we assumed that Raz decays only to Rru, Rru is stable, and there are no other mass losses. Therefore, we assumed *P* = 1.^35^ Under this assumption, the slope of the linear relationship between ln(Rru/Raz + 1) and incubation time since the spike addition of Raz provides a proxy for aerobic MMA.

### Calculation of Potential Carbon Emissions

To assess the extent to which microbial activity associated with streambed fine particles potentially can contribute to C emissions, we applied stream spiraling metrics to calculate in-stream net consumption of organic C (*U* in g C/m^2^/d) in the form of FPOM, calculated as follows
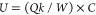
2where *C* is the average carbon areal standing stock measured at *x* = 100 m (FPM × % C/100) to measure the influence of WWTP effluent inputs, *Q* (L/s) is the average stream discharge, *W* (m) is the average width, and *k* is the slope of the exponential decline in % OM of fine particles with distance.^36^ For this approximation, we assume that decline in % OM of FPM along the reach is due to OM consumption, which results in CO_2_ production. Although in-stream CO_2_ production derived from net C removal may not necessarily result in direct CO_2_ emissions, it is highly likely since inland waters are often supersaturated with CO_2_.^3,5^

### Statistical Analysis

We used a Wilcoxon Kruskal Wallis test to examine whether FPM standing stock parameters (FPM, FPOM, % OM, C/N, % C, % N, and MMA) differed between the upstream and downstream sampling sites at *x* = 100 m. Comparisons were made by (i) pooling all data together and (ii) splitting data between the dry periods (*i.e.*, June, July, and November) and wet periods (*i.e.*, February and April). To explore the relation between MMA and FPM standing stock characteristics, we used Spearman correlation tests with data for all upstream and downstream sampling locations and dates pooled together (*N* = 89). We used non-parametric tests because our data set was relatively small and often not normally distributed. In all cases, differences were considered statistically significant if *p* < 0.05.

We examined whether FPM standing stock characteristics at the downstream reach followed consistent longitudinal patterns from the WWTP effluent by applying bivariate linear regression models between FPM standing stock variables (median values) and sampling distance from the WWTP (*i.e.*, *x* = 100, 325, 558, 623, 720, and 830 m). These relationships were assessed for data from the dry and wet periods separately. We also explored the relationship between FPM standing stock variables at the *x* = 100 m downstream sampling site and DF using different bivariate regression models. Regression fits were performed by ordinary least squares, and *r*^2^ was used as the goodness of fit. In all cases, relationships were considered statistically significant if *p* < 0.05, and the best-fit was assessed by comparing *r*^2^ values. Statistical analysis was performed with Matlab software version R2021a (The MathWorks, Inc., Natick, MA, USA).

## Results

### Influence of Hydrological Conditions on the Quantity and Quality of WWTP-Sourced FPM Standing Stocks

The mean particle size of the FPM standing stocks in the study reach was approximately 35 μm (*d*_10_ – d_90_ = 6–100 μm). This size represents the finest fraction of fine particles commonly found in streams (*i.e.*, within the silt/clay Wentworth grain size class^37,38^) and therefore is expected to have a high surface area-to-volume ratio that promotes the colonization of microorganisms.

**Figure 2:**
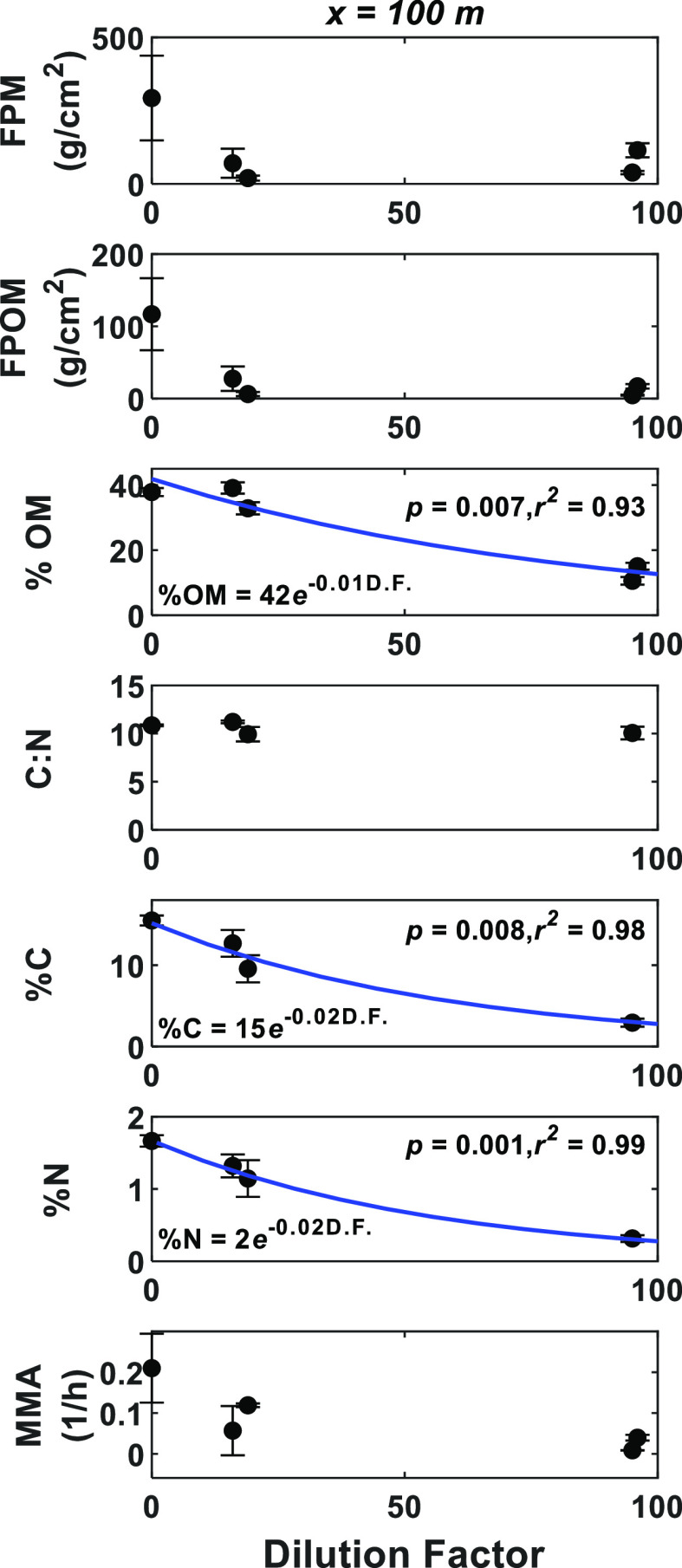
Temporal variability of FPM standing stock characteristics downstream of the WWTP effluent input. Temporal variability is represented by the dilution capacity of the receiving stream at *x* = 100 m downstream of the WWTP source. The DF was <40% during the dry period and >40% during the wet period. Values are medians, and error bars represent the standard error of the mean. For significant relationships, the best-fit equation is shown with a blue line, and the best-fit *p* and *r*^2^ values are shown.

Almost all considered characteristics of the streambed FPM standing stock significantly differed between the locations upstream and downstream (*x* = 100 m) of the WWTP effluent input. Specifically, considering all sampling dates together, FPM, FPOM, and % C were higher downstream of the WWTP effluent, while C/N ratios were lower, and % OM and % N remained similar (Table 1). The spatial changes in FPM characteristics downstream of the WWTP input were mostly observed in the dry period when the impact of the WWTP effluent input on the receiving stream was larger (*i.e.*, lower D.F). In the dry period, all FPM variables showed a significant increase downstream of the WWTP input, except C/N which showed a decrease (Table 1). In addition, variables indicative of FPM standing stock quality (% OM, % C, and % N) decreased substantially with increasing DF (Figure 2).

**Table 1:** Characterization of FPM Standing Stocks in the Streambed Upstream and Downstream at *x* = 100 m of the Input from the Wastewater Treatment Plant Effluent^a^

	all		dry		wet	
	up	down	Up	down	up	down
FPM (g/cm^2^)	36.42 ± 5.54^a^	64.32 ± 40.84^b^	24.86 ± 4.80^a^	70.26 ± 65.42^b^	47.22 ± 8.62^a^	48.34 ± 16.91^a^
FPOM (g/cm^2^)	6.94 ± 1.03^a^	16.88 ± 15.36^b^	6.00 ± 1.25^a^	27.45 ± 23.54^b^	7.80 ± 1.71^a^	6.92 ± 2.74^a^
% OM	19.40 ± 1.48^a^	32.84 ± 3.18^a^	26.68 ± 1.42^a^	37.64 ± 1.12^b^	15.84 ± 1.20^a^	14.04 ± 1.28^a^
C/N	12.58 ± 0.41^a^	10.79 ± 0.31^b^	12.58 ± 0.39^a^	10.85 ± 0.37^b^	11.93 ± 1.09^a^	10.06 ± 0.66^a^
% C	6.43 ± 0.66^a^	9.78 ± 1.52^a^	7.83 ± 0.32^a^	12.69 ± 1.27^b^	3.16 ± 0.39^a^	2.94 ± 0.49^a^
% N	0.64 ± 0.06^a^	1.23 ± 0.16^b^	0.69 ± 0.02^a^	1.38 ± 0.13^b^	0.30 ± 0.02^a^	0.32 ± 0.05^a^
MMA (1/h)	1.88 × 10^–2^ ± 3.4 × 10^–3 a^	5.69 × 10^–2^ ± 3.0 × 10^–2 b^	2.96 × 10^–2^ ± 4.1 × 10^–3 a^	1.22 × 10^–1^ ± 3.9 × 10^–2 b^	1.37 × 10^–2^ ± 3.0 × 10^–3 a^	1.71 × 10^–2^ ± 7.3 × 10^–3 a^

a Values are the medians ± standard error of the mean for all sampling dates together and for dates from dry and wet periods separately. For each variable, a and b indicate statistically significant differences between upstream and downstream locations (Wilcoxon Kruskal Wallis, *p* < 0.05). FPM = fine particulate matter, FPOM = fine particulate organic matter, % OM = percentage organic matter, C/N = carbon to nitrogen molar ratio, % C = percentage carbon, % N = percentage nitrogen, and MMA = microbial metabolic activity.

**Figure 3:**
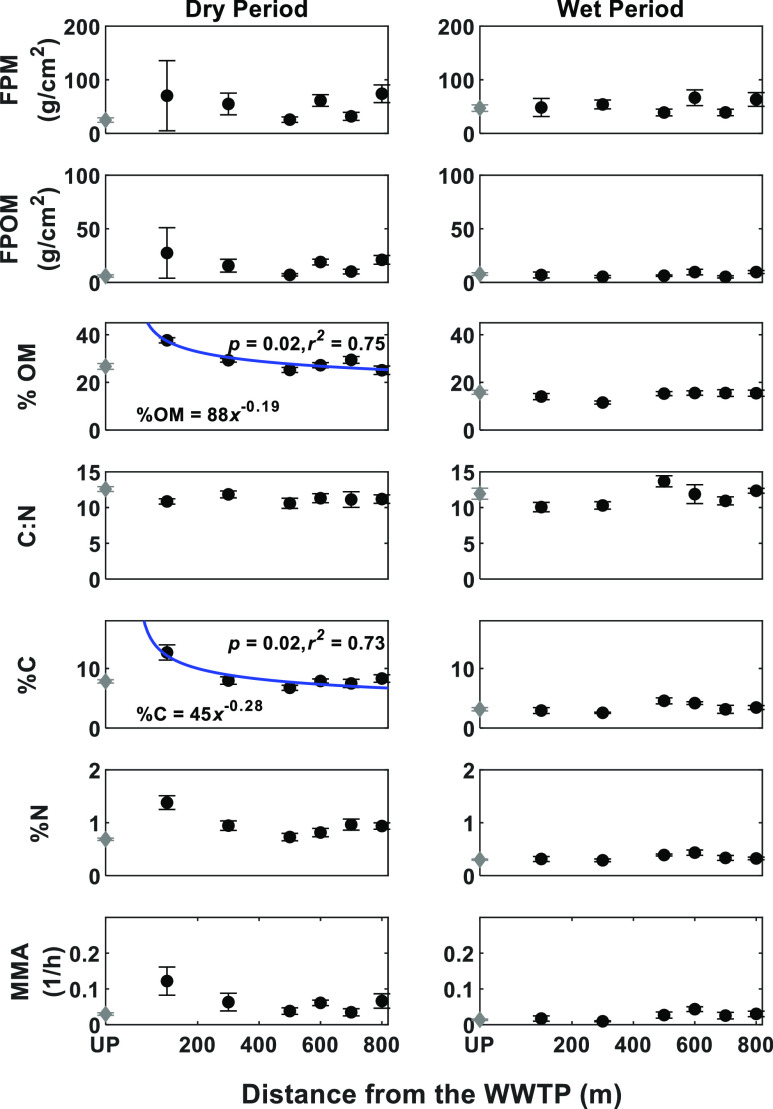
Longitudinal variability of FPM standing stock characteristics downstream of the WWTP effluent input. Longitudinal profiles are shown for the dry (left column) and wet (right column) periods. The upstream value is shown in gray at *x* = UP for upstream, and this sample was taken 800 m upstream of the WWTP outlet. Values are medians, and the error bars represent the standard error of the mean. For significant regressions, the best-fit equation is shown with a blue line, and the best-fit *p* and *r*^2^ values are shown.

During the dry period, % OM and % C decreased with downstream distance from the WWTP input (Figure 3, left column). During the wet period, we did not observe any significant longitudinal pattern with distance from the WWTP input for any of the study variables (Figure 3, right column).

### Microbial Metabolic Activity Associated with FPM Standing Stocks

Rates of MMA measured in resuspended streambed FPM were approximately 1 order of magnitude higher (Table 1) than those measured in the stream water, which averaged 6.1 × 10^–3^ 1/h. This indicates that respiration rates associated with streambed FPM were 9.3 times higher than rates in the water column. Values of MMA associated with streambed FPM were higher downstream (*x* = 100 m) than upstream of the WWTP effluent input during the dry period, whereas no differences were found during the wet period (Table 1). Temporal variation of MMA associated with streambed FPM downstream of the WWTP (*x* = 100 m) was not related to the stream DF (Figure 2). In addition, spatial variability in MMA along the downstream reach did not show any significant trend during the dry or the wet periods (Figure 3).

**Figure 4:**
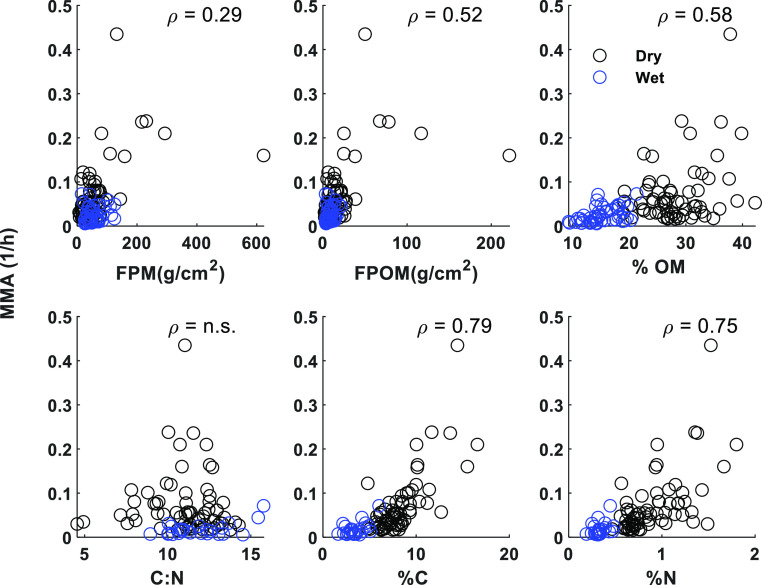
MMA dependence on FPM standing stock characteristics. Relationships between MMA associated with streambed FPM and the quantity (FPM and FPOM) and quality [% OM, carbon (C) and nitrogen (N), and C/N ratio] of the FPM. For significant relationships with *p* < 0.05 considering data from dry and wet periods together, the spearman coefficient (ρ) is indicated. n.s. = not significant.

When data from all the sampling locations were pooled together, we found significant positive relationships between MMA associated with FPM and the quantity of organic particles (*i.e.*, FPM and FPOM); however, the relationship was relatively weak (ρ < 0.52, Figure 4). The relationships between MMA and the quality of FPM were stronger, especially for % C and % N, both indicating higher microbial respiration with higher C and N content in FPM (Figure 4). There was no relationship between MMA and the C/N ratio of FPM.

We estimated that the consumption removal associated with the respiration of OM in FPM standing stocks during the dry period was 3.1 g C/m^2^/d. This value is calculated using the average *Q* and *W* during the dry period (23.6 L/s and 4.93 m, respectively) and assumes an average *C* areal standing stock as measured at *x* = 100 m (FPM × % C/100 = 15.2 g C/m^2^).

## Discussion

### Discontinuities to the River Continuum Modulated by Point Sources and Flow Regimes

Anthropogenic disturbances, such as point sources, alter the expected OM processing signature along the river continuum.^39−41^ Coarser POM is widely known to contribute to overall stream metabolic activity (*e.g.* Tank *et al.*(16)). Here, we focus on the finer size fraction of POM that is sourced by the WWTP effluents as it can also have a direct impact on the metabolism of the receiving streams. Our study agrees with previous work that demonstrates increased fine particle standing stocks and ecosystem respiration immediately downstream of a WWTP effluent.^10^ In our case, these effects were observed at a downstream distance where effluent water was well mixed with stream water (*i.e.*, *x* = 100 m). These previous studies have focused on the physical and biogeochemical influence of fine particles accumulated in the streambed sediment on stream ecosystem functioning only measured close to the WWTP effluent input. Our results shed new light on this topic by exploring transport of these particles further downstream and their potential implications on stream functioning. The transport of POM is controlled by deposition and resuspension processes in the streambed, especially during dry periods when fine particles rapidly deposit in the vicinity of the source.^42^ Quick deposition of particles was previously observed downstream of a WWTP during a particle injection experiment,^43^ further suggesting only short longitudinal downstream transport of particulate material from a point source. Concordantly with this line of thought, we measured increased FPM and FPOM downstream of the WWTP compared to upstream. In addition, our findings also indicate a clear downstream propagation of bioreactive fine particles, with high % N and high respiration, sourced from the WWTP effluent over long distances (∼1 km). This longitudinal pattern is more evident during dry periods when the dilution capacity of the receiving stream is low (Figure 3). Downstream propagation of fine particles can be even higher during wet periods; however, it is hard to track the particles due to the high dilution capacity of the stream under these conditions. Therefore, the deposition of fine particles is followed by a constant reworking in the streambed, likely *via* particle transport through hyporheic flow paths and/or resuspension to the water column and downstream transport.

Besides the dynamics of fine particle transport, there are other environmental drivers that can contribute to increase stream respiration along the downstream reach. Bioturbation can remobilize streambed sediments and increase oxygen levels in the anoxic sediment, potentially stimulating aerobic metabolic activity.^44−46^ This mechanism could be important during the dry period when high temperatures and anoxic conditions prevail.^31^ On the other hand, enhanced presence of dissolved OM sourced from the WWTP, combined with increased temperatures, can foster microbial activity and potentially induce stream anoxia and decreases in aerobic metabolic activity.^20^ We observed increased metabolic activity during the dry periods along the downstream reach, likely as a result of the combination of higher water residence times, temperature, nutrient concentrations, and quantity and quality of FPM standing stocks highly determined by the WWTP inputs. Our results exemplify that a constant urban point source can influence stream physico-chemical characteristics and ecosystem functioning of receiving streams over long distances, especially under low flow conditions.

### Finer Fractions of Particulate Organic Matter from Point Sources can Strongly Influence Stream Bioreactivity

Fine particles sourced from WWTP effluents contribute to the heterogeneous matrix of fine particles deposited within the streambed sediment that is continuously changing due to hydrological, biogeochemical, and microbial processes.^37,47^ During baseflow, the chemical composition (*i.e.*, % OM, % C, and % N) of FPM standing stocks within the sediment can be partially driven by biological activity. As heterotrophic bacteria metabolize the OM fraction of FPM standing stocks, they secrete extracellular polymeric substances, that promote the formation of microbial biofilms. Fine particle deposition in streambeds can be further enhanced by the presence of biofilms.^48−50^ The small size of the fine particles deposited in the streambed in our stream (∼35 μm), and the fact that bacteria preferentially attach to smaller particles with greater surface area, supports the idea of a positive feedback loop between biofilm formation and fine particle deposition. Furthermore, fine particles sourced from urban inputs, especially from WWTP effluents, are more likely colonized by bacteria since they originate from the sludge in the bioreactors. Therefore, these particles form irregularly shaped aggregates that can potentially incorporate detrital plant material and inorganic silt and clay during transport downstream;^47,51^ in addition, they are more prone to enhance development of biofilms once they reach the streambeds.

Ecosystem respiration has been previously related to the amount of benthic OM in Mediterranean and temperate streams.^11,52^ We additionally found that the quality of FPM is an important factor for explaining the metabolic activity associated with fine particles. Our sampling technique and MMA analysis did not separate OM and inorganic sediment and included any refractory C present in the system. Still, we found that MMA associated with FPM standing stocks was directly related to their C and N content. This finding agrees with previous studies where POM decomposition rates and C mineralization increased with POM quality.^53,54^ Moreover, we found that the metabolic activity associated with fine particles in the streambed was 9.3 times higher than that in the stream water column. Our results further support the high bioreactivity associated with the dynamic layer of fine particles in the streambed sediment.^55^ Our study is one of the first attempts to directly relate FPM standing stock characteristics with their associated metabolic activity and to provide a mechanistic understanding of how fine particles can ultimately contribute to in-stream heterotrophic activity and ecosystem respiration.

### Implications of Urban-Sourced Fine Particles on In-Stream Metabolism in the Context of the Global Carbon Cycle

Measurements of FPM standing stocks are the net result from the balance between deposition and removal *via* resuspension or biogeochemical processing in the streambed sediment. This balance is strongly controlled by the hydrological conditions of the stream.^56,57^ We found that the combination of all these physical and biogeochemical processes resulted in a net removal of C associated with FPM along the downstream reach during the dry period, when the DF was low because the % OM and % C decreased. This in-stream net C removal can partially be explained by biogeochemical processing of C in between subsequent resuspension events that contributed to further redistribute fine particles within the streambed sediment in the downstream direction. This is further supported by the observed MMA associated with FPM, which is indicative of microbial respiration. The CO_2_ emissions derived from our C removal estimates as driven by respiration associated with streambed FPM during the dry period (3.1 g C/m^2^/d) are similar to values reported in small–medium Swiss streams and rivers receiving WWTP effluent inputs (∼2.5 g C/m^2^/d).^20^ Moreover, our results also fall within the range of CO_2_ emissions estimated over a 20 year time-series study downstream of a WWTP effluent in the Oria River, Spain.^12^ Since our method isolated fine particles deposited in the streambed sediment, the similarity between our measurements and previous reported values suggest that C consumption or respiration associated with standing stocks of bioreactive fine particles can have the potential to largely contribute to overall C emissions to the atmosphere, especially when particles from urban sources are relevant.

Urban waste affects biogeochemical cycles in freshwaters from local to global scales,^18^ with greater potential impacts in areas with water scarcity. Spain has one of the lowest ranges of DFs,^58^ and thus, streams from this region have a high vulnerability to inputs from urban point sources. However, intermittent streams are prevalent worldwide. We directly observed how the accumulation of urban-sourced bioreactive fine particles led to increased respiration, which is exacerbated during the dry period. The longitudinal footprint of urban-sourced bioreactive fine particles may continue to evolve in space and time as longer dry periods from increased urbanization and climate change are expected. These conditions would have direct implications to stream metabolism and potentially enhanced CO_2_ emissions to the atmosphere. Therefore, water scarcity combined with discontinuities to the river continuum caused by urban point sources can result in hot spots of aerobic respiration during dry periods, with potentially long-term implications for global carbon cycles.
